# “Men’s health – a little in the shadow”: a formative evaluation of medical curriculum enhancement with men’s health teaching and learning

**DOI:** 10.1186/s12909-015-0489-9

**Published:** 2015-11-26

**Authors:** Carol A. Holden, Veronica R. Collins, Christopher J. Anderson, Sylvia Pomeroy, Richard Turner, Benedict J. Canny, Bu B. Yeap, Gary Wittert, Rob I. McLachlan

**Affiliations:** 1 Andrology Australia, c/o School of Public Health and Preventive Medicine, Monash University, Prahran, VIC Australia; 2 School of Public Health and Preventive Medicine, Monash University, Prahran, VIC Australia; 3 School of Medicine, University of Tasmania, Hobart, Australia; 4 Faculty of Medicine, Nursing and Health Sciences, Monash University, Victoria, Australia; 5 School of Medicine and Pharmacology, University of Western Australia, and Department of Endocrinology and Diabetes, Fremantle and Fiona Stanley Hospitals, Perth, WA Australia; 6 Freemasons Foundation Centre for Men’s Health, University of Adelaide and Endocrine and Metabolic Unit, Royal Adelaide Hospital, Adelaide, South Australia Australia; 7 Hudson Institute of Medical Research, Clayton, Victoria and Monash IVF, Richmond, VIC Australia; 8 Andrology Australia, c/o School of Public Health and Preventive Medicine, Monash University, PO Box 315, Melbourne, VIC 3004 Australia

**Keywords:** Men’s health, Medical education, Curriculum enhancement, Implementation, Community of practice

## Abstract

**Background:**

Enhancing a medical school curriculum with new men’s health teaching and learning requires an understanding of the local capacity and the facilitators and barriers to implementing new content, and an approach that accommodates the systemic and cultural differences between medical schools.

**Methods:**

A formative evaluation was undertaken to determine the perspectives of key informants (academics, curriculum developers) from four Australian medical schools about the strategies needed to enhance their curriculum with men’s health teaching and learning. Through semi-structured questioning with 17 key informants, interviewees also described the contextual barriers and facilitators to incorporating new topic areas into existing curriculum. Interviews were recorded with consent, transcribed verbatim, and analysed by two researchers to identify key themes.

**Results:**

Interviewees were enthusiastic about incorporating men’s health content through a men’s health curriculum framework but highlighted the need for systems to assist in identifying gaps in their current curriculum where the men’s health topics could be integrated. The student experience was identified as a key driver for men’s health teaching and learning. Furthermore, core men’s health clinical outcomes needed to be defined and topic areas vertically integrated across the curricula. This would ensure that students were appropriately equipped with the skills and knowledge for subsequent clinical practice in a range of geographical settings. Interviewees consistently suggested that the best implementation strategy is to have someone ‘on the ground’ to work directly with medical school staff and champion the men’s health discipline. Providing mechanisms for sharing knowledge and resources across medical schools was highlighted to facilitate implementation, particularly for those medical schools with limited men’s health teaching resources.

**Conclusions:**

Despite the unanimous support for men’s health teaching and learning, the evaluation highlighted that the student experience must be recognised as paramount when integrating new topic areas into an already packed curriculum. A community of practice, where medical schools share relevant resources and knowledge, could help to ensure a commonality of student experience with respect to men’s health learning in medical schools across different geographical settings and with different levels of resourcing. Such an approach could also be adapted to other areas of curriculum enhancement.

## Background

There are many pressures on medical schools to adapt their curriculum to ensure that medical students are being trained as future doctors [1]. Drivers of this imperative include rigorous standards from regulatory agencies, increased popularity of integrated curricula, a rapid expansion of knowledge and workforce pressures [1, 2]. However, these drivers create tension between the need for change and the complexity of medical curriculum, with a range of barriers that inhibit curricular change despite the best intentions [3]. Moreover, systemic and cultural differences between medical schools has led to national curriculum frameworks being implemented to varying degrees, according to the local setting and administrative processes [4]. Enhancing a medical school curriculum to respond to changing healthcare and population needs requires an understanding of the local capacity to implement new content, and the factors that facilitate or inhibit the implementation process. However, as Kerkering and Novick [5] note, with reference to the integration of a population health framework, there is a greater need to identify the process of implementation rather than merely describing the contextual enablers and barriers to change. Importantly, understanding those implementation strategies that have demonstrated improvements in teaching and learning, irrespective of the topic area, also provide potential evaluation frameworks to further advance knowledge in the field [6].

Over recent years there has been greater recognition of men’s health issues in many countries [7], with several releasing policy documents and strategic frameworks to improve the disparities seen amongst different groups of men [8]. The Australian Government released a National Male Health Policy that includes explicit calls to action by universities and training organisations to improve workforce capacity in male health [9]. Indeed, studies have shown that doctors can lack confidence in managing men’s health, particularly sexual and reproductive health [10, 11]. Furthermore, medical students report a perceived lack of preparedness for men’s health clinical practice, with a need to enhance men’s health education in Australian medical schools [12]. However, despite some work in the USA [13] and Australia [14] on teaching urology topics, the broader inclusion of male health in the medical school curriculum is not well developed. To address this need, several Australian medical schools have worked to develop a draft male health curriculum framework that includes a range of men’s health topics that could be incorporated into existing medical school curriculum.

A formative evaluation was undertaken to explore the local capacity and the potential constraints for Australian medical schools to integrate the framework for men’s health teaching and learning into their existing curricula. The evaluation focused on identifying implementation strategies that could be applied across different medical schools to maximise the opportunity for a common learning experience in men’s health for students across Australia.

## Methods

A qualitative study was undertaken to elicit the views of key informants (senior management, medical educators and curriculum developers) about their perceptions of the value of including men’s health teaching and learning in Australian medical schools and the barriers and facilitators to incorporating new content generally, and the men’s health content specifically, into existing curriculum. Strategies and resources needed to support the medical schools in implementing men’s health teaching and learning were also explored.

### Study participants

Four Australian medical schools delivering undergraduate medical degrees participated in the formative evaluation. The key informants in the evaluation included:

- Senior managers with governance responsibilities and drivers of educational reform from each of the selected universities, e.g., Deans of Medical Schools
- Clinical educators from hospital and community settings
- Academic staff with experience in curriculum design and/or experience with initiatives that enhance student learning, e.g., chairpersons of medical education committees, curriculum designers, coordinators of curriculum development, unit coordinators.

A snowball sampling approach identified key informants at each participating medical school.

### Data collection and analysis

Semi-structured interviews took place at mutually convenient times at each participating medical school. Through open-ended questioning, interviewees provided a personal perspective on integrating men’s health teaching and learning into existing medical school curricula. The following domains of interest were explored: i) the perceived level of interest in men’s health teaching and learning; ii) the barriers and iii) the enablers to integrating teaching and learning in men’s health; and iv) the potential implementation strategies (systems and resources) required to support the integration of men’s health teaching and learning into current curricula. With participant consent, interviews were audio-taped, and transcribed verbatim. Two investigators (CH and VC) undertook a thematic analysis of informant transcripts using an inductive approach [15]. Within the broad domains of enquiry (i-iv above), data were coded and themes identified across cases by each of the two investigators. Themes were then compared and consensus reached through discussion. Direct quotes from participants are reported to illustrate the themes.

### Ethics approval

Ethics approval was given by the Monash University Human Research Ethics Committee, Melbourne, Australia (CF13/2393 - 2013001264). The Social Science Human Research Ethics Committee of the University of Tasmania (no. H0013535) gave secondary approval.

## Results

Seventeen interviews were conducted between October and November 2013. A breakdown of the participants by medical school is summarised in Table 1.

**Table 1:** Participant details

University	Key informant and Academics (N)				
	Academic executives^a^	Hospital based clinicians^b^	Community-based clinicians^c^	Curriculum quality academics^d^	Total Individual Interviews
University 1 (UNI 1)	1	1	0	1	3
University 2 (UNI 2)	1	1	3	2	7
University 3 (UNI 3)	2	0	0	2^e^	4
University 4 (UNI 4)	1	1	0	1	3
Total number	5	3	3	6	17

^a^Academic executives: Deans, Heads of School, Directors of Medical Education

^b^Hospital based clinicians: clinicians in hospitals and working for the university

^c^Community based clinicians: clinicians in community practice and working for the university

^d^Curriculum quality academics: Academics in quality and mapping

^e^No representative from curriculum mapping

### Men’s health teaching and learning in the curriculum

Despite a range of views on what might constitute a men’s health curriculum, most participants confirmed that presently men’s health in general, and male-specific topics (e.g., genital examination) in particular, were under-represented in Australian medical curricula. Some used women’s health as the benchmark and others thought the lack of focus on men’s health reflected broader societal attitudes.

*“I think certainly that men’s health is an area that perhaps has laid a little in the shadow.“[UNI-3-4]*

However, there was recognition that men’s health content should not be included at the expense of women’s health.

*“..make sure it is equivalent in the pre-clinical years with men and women, not equal but equivalent because they are not the same.” [UNI-1-2]*

### Barriers to implementing men’s health teaching and learning

There was wide ranging discussion on the barriers to implementing men’s health teaching and learning. Participants highlighted both barriers to the inclusion of any new content into existing curricula as well as specific barriers relevant to men’s health teaching.

### Pressures from outside the medical school

Many participants reported that pressures on medical schools from external interests to incorporate new material into already full curricula resulted in ever-increasing resourcing pressures and potential student overload.

*“…a continual desire by individuals and groups to bring new material into the curriculum.” [UNI-2-2]*

Similarly, it can be difficult for medical schools to incorporate new topic areas given there is no standard way to integrate new content across the medical school curriculum.

*“…in the UK there is a GMC [General Medical Council] curriculum framework that all the medical schools adhere to. …you don’t want this framework and then the musculoskeletal framework and we also have the AMC [Australian Medical Curriculum] framework that we have to do…they need to stick together” [UNI-2-4]*

### Internal resistance to change

Participants noted that as each university medical school is embedded within layers of internal governance and organisational culture, barriers to change can occur if the level of bureaucracy required to implement change becomes overwhelming.

*(We need)“... an efficient way so that you are not bogged down by too much bureaucracy” [UNI-2-3]*

Organisational and individual resistance to change can further impede navigation through complex systems.

*“…many other barriers that come up that can be quite idiosyncratic just based on people who don’t want to change anything anytime irrespective of whether it is good, bad or indifferent” [UNI-2-2]*

Some suggested that the process for including new curriculum frameworks was not clear or systematic.

*“The locus of control for the curriculum isn’t clear. There isn’t a curriculum committee…..it’s negotiations with individual people” [UNI-1-2]*

### Lack of workforce capacity

Workforce resourcing was identified as a potential barrier, including: lack of teaching staff expert in the area, including relevant specialists for clinical placements; little time to devote to implementing new topic areas; and lack of staff dedicated to mapping curriculum to manage the insertion of new material. Men’s health was perceived to be a topic area where it may be more difficult to find experts, particularly for clinical placements.

*“There is a lot of need for teaching across the entire University and a limited pool of people from which have the requisite skills and background to do those types of teaching” [UNI-3-4]*

*“So we have tried to include the content or make it better taught but, I mean all medical school have these sort of areas…some areas are traditionally difficult to find teachers and this is one of them for us.” [UNI-2-4]*

### Finite ‘crowded’ curriculum and curriculum complexity

Many comments pertained to the logistical and resourcing challenges of adding new curriculum frameworks, such as finding space in an already crowded curriculum and the complexity of identifying how and where new topics should be incorporated into the overarching structure.

*“very challenging because people like to have their new ideas put in and very rarely do people who are already doing something wish to have their existing or assessment removed” [UNI-2-2]*

### Lack of a home for ‘men’s health’

In addition to general barriers to curriculum change, a men’s health curriculum raised some specific issues. Several participants suggested that as ‘men’s health’ does not belong to an established discipline, there is a lack of ownership of the subject area with no single department advocating for its inclusion.

*“I think that, at … We’ve struggled to locate a space for male health. … part of the reason we struggle is that women’s health is, …recognized disciplines that you can go to, like obstetrics and gynaecology, and the fall-back for male health seems to be … urology….So if we’re going to have a male health component where does it sit? Who’s responsible for it? … it shouldn’t just be ‘put in general practice ‘cause it doesn’t sit anywhere else’ sort of thing but then, who will champion it?”[UNI-1-3]*

### Enablers to implementing men’s health teaching and learning

Along with the discussion of barriers, participants offered ideas for how these could be overcome, as well as suggesting other enablers to implementation of new curriculum in general, and men’s health in particular.

### Champions

Participants consistently noted that curriculum change is greatly facilitated by advocates (‘*champions*’) who push for change. Thus, identifying local champions – those with passion and a vested interest – was considered vital to demonstrate the relevance of men’s health teaching and learning. They can also facilitate integration into current teaching and provide leadership and a more structured approach to the implementation process.

*“Engage with the local champions is probably the most important thing. So someone can push it and make it relevant. … It just can’t be something that’s tacked on the side as an extension. It’s got to integrate smoothly with the curriculum and that’s difficult because every curriculum is designed differently.” [UNI-2-5]*

However, it was also noted that there needs to be broader support, importantly from high-level decision-makers, and possibly from local clinicians, before implementation would happen.

*“…introducing contemporary, best practice curriculum when it is promoted and supported by clinicians who are well represented and well recognized and competent in the marketplace. “[UNI-2-3]*

Similarly, sustainability was identified as a factor in the overall success of any implementation process, which would be at risk when relying on a single champion to implement the curriculum framework.

*“… but ideally, there needs to be either two champions or there has to be a men’s health unit. Something behind that keeps driving it, for when the champion moves on to the next job” [UNI-4-1]*

### External advocacy

Many participants suggested that external advocacy efforts in raising the awareness of men’s health within medical schools would support advocacy by local champions and provide some credibility for those teaching the curriculum.

*“in a sense curriculum marketing, if I could use that word (laughs), to make sure that people are on side to see the value of this” [UNI-3-2]*

Participants suggested that the delivery of men’s health teaching and learning could be supported through multiple levels across the broader external community. Firstly, independent men’s health advocacy groups could play a role in supporting the implementation of the men’s health curriculum, primarily through the provision (and update) of teaching resources that would enhance the learning experience and delivery.

*“I don’t know whether it might be more useful to have these resources as online links perhaps or something like that that could be then periodically reviewed by [men’s health advocacy groups]. That’s a fairly big logistical task but I appreciate that.” [UNI-3-4]*

Some participants also suggested that external men’s health organisations may have a role in actively participating with medical schools to provide human resources and education expertise if local champions or specialists were limited (or lacking). Furthermore, participants suggested that a ‘*community of practice*’ could be fostered that would enable the sharing of ‘*innovations and ideas’* across participating medical schools.

*“I guess you know you can certainly do a lot of that, if you’ve got a local champion you could build up a virtual community of practice where people can share innovations and ideas.” [UNI-2-5]*

### Windows of opportunity

Although some participants noted that changes in the external environment, specifically the current move from undergraduate to graduate courses that was happening in many universities in Australia at the time, was a potential barrier, such external changes may also present as windows of opportunity for the implementation of new material generally and the men’s health curriculum specifically.

“*well the time is right for us because we are changing our curriculum”…”So if we weren’t changing our curriculum then it would be less relevant” [UNI-4-1]*

### Potential implementation strategies

Participants discussed a range of strategies that could facilitate the development and implementation of new material, in this case men’s health, into the current medical curriculum. There was a consistent underlying theme that in this process, student learning needs should be paramount and implementation will require student-focused strategies.

### Active learning

Medical students were often described as ‘*active learners*’ and proactive in advancing their education, despite a tendency for assessment to drive learning, particularly in pre-clinical years.

*“..assessment has to be at the heart of learning… these kids respond to assessment” [UNI-1-2]*

Participants also noted however that assessment was not the only driver, with students being better engaged when they appreciate the relevance and application of the learning.

*“… engage medical students is when they see people who do the area that they are talking about…they see the clinical relevance of it …inspire them about aspects of male health is by seeing someone who does those aspects of male health.” [UNI-4-3]*

### Supplementing clinical placements

Some participants noted that as students were geographically dispersed across different clinical placements, some consideration needed to be given to the mode of delivery, particularly when the teaching resources may be limited. Digital technologies and online delivery of men’s health content was identified as an alternative strategy to support clinical placements, which vary within and between medical schools.

*“…because of their placements they are usually dispersed everywhere. If we were going to do something to the whole group either that could be delivered wherever they are at the time or delivered by technology.”[UNI-2-1]*

Several participants suggested that online delivery of specific modules is particularly relevant during clinical teaching years, potentially maximising the student learning experience and addressing a lack of consistency in the material to which students are exposed.

*“..to add any more didactic type teaching into that clinical program is very difficult. It is perceived as compromising the direct work experience that students would get.“[UNI-2-1]*

### Managing sensitive topics in diverse student populations

Some participants noted that sensitivity and understanding was needed with the teaching of some areas of men’s health, as for women’s health, due to the diversity in the student population with respect to age, gender, culture and religious beliefs.

*“We have women from cultural groups who, for things like contraception, sexual transmitted diseases, even sexual responses, male and female sexual responses, are incredibly challenging lectures to sit in …we ignore that at our peril. We have to explicitly understand the cultural…“[UNI-1-2]*

Furthermore, participants at one medical school indicated that clinical placements could be supplemented with a dedicated program of volunteers (Clinical Teaching Associates) for male (and female) examinations that would support students in being more comfortable and less embarrassed when performing sensitive examinations.

*“…when you start to get to more intimate stuff, for both males and females, it becomes more difficult to find patients who are willing to let students examine them, and it becomes more difficult for the students themselves to sort of deal with that, because it’s a sensitive area, they’re young, they’re dealing with older people,...“[UNI-1-3]*

### Flexibility

Acknowledging that not all students will have a major interest in men’s health, some participants suggested that the framework should be flexible, to provide opportunities for interested students to explore a men’s health elective during clinical placements, for example.

*“If there is a particular student(s) who does have an interest in men’s health there would be some flexibility to pursue that interest in an elective type format or a choice format in those clinical years.“[UNI-3-4]*

Participants agreed that the imperative for all medical schools is to ensure that students received a uniform but diverse experience. One participant reflected on the benefits of a defined male health curriculum framework as providing:

*“..a commonality of curriculum experience. So I would like to think that maybe certain areas of content would be covered everywhere but the nature of the … delivery might be a little bit different.“[UNI-2-1]*

## Discussion

This evaluation study of medical school key informants identified a number of implementation strategies and explored the barriers and enablers to men’s health teaching and learning in the current medical school curriculum. For medical schools, a strategy of 'curriculum enhancement' has been suggested as a more feasible alternative to curriculum expansion or substitution of a distinct course [5]. The pressures on medical schools to adapt to a changing environment are well recognised [14]. Indeed, Bordage and Harris [16] identified five key elements that contribute to these pressures: the expected competencies and roles; the learners at the centre; assessment linking competencies and learners; the conditions and resources for learning; and a multifaceted socio-political cultural context in which the learning occurs, which were confirmed in this evaluation. Our findings highlight some student-focused implementation strategies to address these pressures and progress curriculum enhancement in men’s health that can be adapted to other curriculum areas.

Others have identified enablers of curriculum enhancement, including: the existence of a defined curriculum framework; establishment of a trusted and shared agenda with medical course directors and key stakeholders [5]; identifying faculty champions; supporting student initiatives for inclusion; and supporting interprofessional development [17]. Our findings support the importance of champions and organisational structures to address the teaching, workforce and resource barriers to implementation, allowing more effective enhancement of medical school curricula.

Understanding local capacity and the specific administrative and cultural environment is vital to integrating new content into existing curricula at the local level. The implementation phase of curriculum change can be protracted despite the in-principle support for new frameworks [4]. However, as demonstrated by the Leaders in Indigenous Medical Education (LIME) Network, established to support the implementation of an Indigenous Health Framework in Australian medical schools, strategies can be developed over time that encourage and support implementation [18]. While this study demonstrates that medical schools support the notion of men’s health learning, an innovative approach to curriculum enhancement may be required to minimise the potential resourcing barriers to implementation. Importantly, implementation strategies identified in this evaluation focused primarily on enhancing the student learning experience because such a focus tends to remove the discipline specific nature of curriculum enhancement.

As proposed by Mazel and Ewen [6] when evaluating the LIME Network, a community of practice (CoP) can be an effective way to facilitate curriculum enhancement with men’s health teaching and learning and could address some of the barriers and enablers identified in this, and other studies. CoPs can provide opportunities for educators with shared interests (‘champions’) to navigate the internal structures and processes and share their experience to create new knowledge and change within the curriculum [19]. Wenger [20] defined CoPs as “groups of people who share a common concern, a set of problems, or a passion about a topic, and who deepen their knowledge and expertise in this area by interacting on an ongoing basis”. Wenger [21] further described three fundamental elements of a CoP: joint enterprise (what it is about, ‘domain’); mutual engagement (the interactions that lead to shared meaning, ‘community’); and a shared repertoire (the resources such as techniques, experiences or process, ‘practice’). CoPs have gained recognition in healthcare and health professions [19, 22–24], in higher education [25], and in professional development [26]. McDonald [27] identified the benefits of a CoP in education, also noting that effective facilitation is needed to allow an established CoP to thrive, albeit her study was conducted at only one university. In the context of men’s health teaching and learning, extending the CoP to include men’s health stakeholders would potentially assist in overcoming workforce and resource barriers for longer-term sustainability. Furthermore, the potential to share resources and knowledge as part of a CoP could help to ensure a ‘commonality of student experience’ across Australian medical schools, an enabler highlighted in this study. Promoting medical schools that have successfully undergone curriculum enhancement as 'Lighthouse' exemplars, and partnering with outside agencies with subject expertise and 'buy-in' resources have previously been identified as effective ways for medical schools to cooperate to support curriculum enhancement [17]. This approach can be readily adopted for curriculum enhancement in the area of men’s health, as well as other curriculum areas where external stakeholders are willing, and able, to support medical curricula.

One of the challenges of a CoP identified by a number of authors, albeit of large scale, national, multi-organisational and multi-professional CoPs [22–24, 28], is devising an appropriate evaluation framework. All authors identify two primary challenges as i) lack of a conceptual framework and the moving from theory to practice and ii) identifying outcome (in this case, the inclusion of men’s health teaching and learning in existing medical curricula) and process measures. Fung-Kee-Fung [22–24, 28] developed a framework for process and outcome evaluation of CoPs that was utilised and tested by Mazel and Ewen [6] for the evaluation of the LIME Network. Another important consideration, as identified by Fung-Kee-Fung [22–24, 28] and supported in this study and an evaluation of the LIME Network [6], is that a CoP requires a range of supportive infrastructure tools for its success: project management, communication strategies and access to evidence. Process evaluation will be important to identify the enablers and barriers to facilitating knowledge sharing and relationships in the CoP for translation into best practice pedagogy for teaching and learning in men’s health.

The perceived window of opportunity provided by the current medical curriculum restructure in many universities, as they move to graduate courses, may add to the momentum to improve men’s health teaching in Australian medical schools. However, our findings suggest that the standing of men’s health in the community may be a bigger barrier to adopting enhanced men’s health training for medical students than apparent disinterest within medical schools. Australia is one of only two countries in the world to have defined a men’s health policy [9, 29], despite many countries recognising the need [30]. In Australia, some changes are evident, with increased community awareness of men’s health issues such as prostate cancer, testosterone supplementation, erectile dysfunction, social isolation and domestic violence. It is vital that the current and future medical workforce is appropriately trained to respond to the changing external environment and population health needs.

Furthermore, while government and/or specialist groups may advocate the need for men’s health teaching, the learning experience of the student is paramount [3]. Ensuring that clinically-relevant men’s health teaching is an overt part of medical training is vital to develop the student’s interest and preparedness in men’s health practice, a perspective supported by medical students [12]. Indeed, Powell and colleagues [31] demonstrated that the number of sensitive examinations (male and/or female) performed is a predictor of student confidence. Yet, students generally report variability in the opportunities for sensitive male examinations, which was markedly different to female examinations where opportunities existed over several years of the course [12]. A defined yet flexible curriculum framework can help to support a ‘commonality of experience’ for students, reduce variability in learning opportunities between students and provide adequate preparation for clinical practice.

### Study limitations

Medical schools participating in this evaluation were those with representatives on the project Working Group that was overseeing the development of a draft men’s health curriculum framework. Consequently, a study limitation is the potential bias towards men’s health learning and teaching that may not be evident in other medical schools. The findings from this evaluation may therefore not be generalizable beyond the current study participants and schools interviewed; however, this study will inform an implementation strategy to be developed and piloted at these and other medical schools, with corresponding evaluation and data collection. Despite the limitations, however, the themes identified from these key informants were consistent with those identified in the literature.

## Conclusion

A climate of readiness for change was identified in this formative evaluation, with participating medical schools expressing enthusiasm for defined men’s health teaching and learning through a student-focused implementation approach. While men’s health was the focus of this evaluation, the proposed community of practice approach, evaluation framework and implementation strategies identified are not specific to any single discipline. By focusing on student learning, the discipline-specific nature of implementation strategies is removed.
